# Supplementary material for: Modeling glioblastoma heterogeneity as a dynamic network of cell states
Source: Mol Syst Biol. 2021 Sep 16;17(9):e10105. doi: 10.15252/msb.202010105 (PMC8444284; doi:10.15252/msb.202010105)
Supplement: Supplementary file 5 — Source Data for Figure 3 [file MSB-17-e10105-s001.zip › Figure3A_sourcedata/GSEA_3065/hallmarks_state6.GseaPreranked.1623416308702/HALLMARK_IL2_STAT5_SIGNALING.html]

Details for gene set HALLMARK\_IL2\_STAT5\_SIGNALING[GSEA]

|  || Dataset | state6 |
| Phenotype | NoPhenotypeAvailable |
| Upregulated in class | na\_neg |
| GeneSet | HALLMARK\_IL2\_STAT5\_SIGNALING |
| Enrichment Score (ES) | -0.35103062 |
| Normalized Enrichment Score (NES) | -1.413123 |
| Nominal p-value | 0.027734976 |
| FDR q-value | 0.074777514 |
| FWER p-Value | 0.639 |
Table: GSEA Results Summary

  

Fig 1: Enrichment plot: HALLMARK\_IL2\_STAT5\_SIGNALING      
 Profile of the Running ES Score & Positions of GeneSet Members on the Rank Ordered List

  

| PROBE | GENE SYMBOL | GENE\_TITLE | RANK IN GENE LIST | RANK METRIC SCORE | RUNNING ES | CORE ENRICHMENT || 1 | CCND2 |  |  | 32 | 0.269 | 0.0338 | No |
| 2 | ODC1 |  |  | 222 | 0.169 | 0.0356 | No |
| 3 | RGS16 |  |  | 239 | 0.164 | 0.0566 | No |
| 4 | NCS1 |  |  | 362 | 0.141 | 0.0623 | No |
| 5 | GPX4 |  |  | 365 | 0.141 | 0.0817 | No |
| 6 | HIPK2 |  |  | 445 | 0.128 | 0.0906 | No |
| 7 | PHLDA1 |  |  | 612 | 0.113 | 0.0872 | No |
| 8 | DENND5A |  |  | 920 | 0.090 | 0.0645 | No |
| 9 | ITGAE |  |  | 962 | 0.088 | 0.0720 | No |
| 10 | AHCY |  |  | 1084 | 0.081 | 0.0694 | No |
| 11 | RRAGD |  |  | 1144 | 0.078 | 0.0735 | No |
| 12 | GLIPR2 |  |  | 1295 | 0.071 | 0.0662 | No |
| 13 | IFITM3 |  |  | 1398 | 0.068 | 0.0639 | No |
| 14 | KLF6 |  |  | 1424 | 0.067 | 0.0703 | No |
| 15 | NDRG1 |  |  | 1488 | 0.065 | 0.0721 | No |
| 16 | GADD45B |  |  | 1497 | 0.064 | 0.0801 | No |
| 17 | SYNGR2 |  |  | 1606 | 0.060 | 0.0761 | No |
| 18 | SOCS2 |  |  | 1611 | 0.060 | 0.0840 | No |
| 19 | SWAP70 |  |  | 1657 | 0.058 | 0.0869 | No |
| 20 | MAP6 |  |  | 1689 | 0.057 | 0.0914 | No |
| 21 | CDKN1C |  |  | 1788 | 0.054 | 0.0876 | No |
| 22 | MAPKAPK2 |  |  | 1804 | 0.053 | 0.0933 | No |
| 23 | BHLHE40 |  |  | 1880 | 0.051 | 0.0918 | No |
| 24 | BATF3 |  |  | 2047 | 0.046 | 0.0792 | No |
| 25 | MYC |  |  | 2081 | 0.045 | 0.0818 | No |
| 26 | SLC2A3 |  |  | 2103 | 0.045 | 0.0856 | No |
| 27 | BCL2L1 |  |  | 2164 | 0.043 | 0.0847 | No |
| 28 | ANXA4 |  |  | 2272 | 0.039 | 0.0778 | No |
| 29 | SNX9 |  |  | 2477 | 0.034 | 0.0591 | No |
| 30 | MYO1C |  |  | 2614 | 0.031 | 0.0477 | No |
| 31 | CASP3 |  |  | 2634 | 0.030 | 0.0498 | No |
| 32 | AHR |  |  | 2711 | 0.029 | 0.0450 | No |
| 33 | COCH |  |  | 2788 | 0.027 | 0.0401 | No |
| 34 | NCOA3 |  |  | 3017 | 0.022 | 0.0169 | No |
| 35 | GABARAPL1 |  |  | 3023 | 0.022 | 0.0193 | No |
| 36 | DCPS |  |  | 3126 | 0.019 | 0.0103 | No |
| 37 | ARL4A |  |  | 3127 | 0.019 | 0.0130 | No |
| 38 | SLC39A8 |  |  | 3275 | 0.016 | -0.0017 | No |
| 39 | SPRED2 |  |  | 3498 | 0.011 | -0.0257 | No |
| 40 | HUWE1 |  |  | 3751 | 0.006 | -0.0539 | No |
| 41 | CDC42SE2 |  |  | 3771 | 0.005 | -0.0553 | No |
| 42 | APLP1 |  |  | 3813 | 0.004 | -0.0594 | No |
| 43 | ENPP1 |  |  | 3916 | 0.002 | -0.0709 | No |
| 44 | IGF1R |  |  | 3930 | 0.002 | -0.0721 | No |
| 45 | CCND3 |  |  | 4016 | 0.000 | -0.0819 | No |
| 46 | SPRY4 |  |  | 4103 | -0.001 | -0.0916 | No |
| 47 | LRIG1 |  |  | 4165 | -0.002 | -0.0982 | No |
| 48 | SYT11 |  |  | 4256 | -0.004 | -0.1080 | No |
| 49 | XBP1 |  |  | 4331 | -0.006 | -0.1157 | No |
| 50 | CSF1 |  |  | 4350 | -0.006 | -0.1170 | No |
| 51 | EEF1AKMT1 |  |  | 4384 | -0.007 | -0.1199 | No |
| 52 | TIAM1 |  |  | 4472 | -0.008 | -0.1287 | No |
| 53 | PLIN2 |  |  | 4478 | -0.008 | -0.1281 | No |
| 54 | FAM126B |  |  | 4493 | -0.009 | -0.1286 | No |
| 55 | PRAF2 |  |  | 4511 | -0.009 | -0.1293 | No |
| 56 | ITGAV |  |  | 4722 | -0.013 | -0.1516 | No |
| 57 | UCK2 |  |  | 4845 | -0.015 | -0.1635 | No |
| 58 | CAPG |  |  | 4878 | -0.016 | -0.1649 | No |
| 59 | PTRH2 |  |  | 4884 | -0.016 | -0.1632 | No |
| 60 | CCNE1 |  |  | 4890 | -0.016 | -0.1615 | No |
| 61 | FURIN |  |  | 4962 | -0.017 | -0.1673 | No |
| 62 | RNH1 |  |  | 4982 | -0.018 | -0.1670 | No |
| 63 | PTCH1 |  |  | 5310 | -0.025 | -0.2011 | No |
| 64 | CA2 |  |  | 5950 | -0.037 | -0.2695 | No |
| 65 | UMPS |  |  | 6019 | -0.039 | -0.2719 | No |
| 66 | PLSCR1 |  |  | 6134 | -0.041 | -0.2793 | No |
| 67 | PDCD2L |  |  | 6295 | -0.044 | -0.2915 | No |
| 68 | ST3GAL4 |  |  | 6452 | -0.049 | -0.3027 | No |
| 69 | CDC6 |  |  | 6482 | -0.049 | -0.2991 | No |
| 70 | POU2F1 |  |  | 6515 | -0.050 | -0.2958 | No |
| 71 | FAH |  |  | 6682 | -0.055 | -0.3073 | No |
| 72 | HOPX |  |  | 6940 | -0.063 | -0.3280 | No |
| 73 | TNFRSF21 |  |  | 7096 | -0.068 | -0.3364 | No |
| 74 | PLEC |  |  | 7224 | -0.072 | -0.3410 | Yes |
| 75 | ETV4 |  |  | 7263 | -0.073 | -0.3352 | Yes |
| 76 | BMP2 |  |  | 7299 | -0.074 | -0.3288 | Yes |
| 77 | RHOB |  |  | 7306 | -0.075 | -0.3191 | Yes |
| 78 | PLPP1 |  |  | 7391 | -0.078 | -0.3180 | Yes |
| 79 | WLS |  |  | 7516 | -0.082 | -0.3208 | Yes |
| 80 | PHTF2 |  |  | 7595 | -0.086 | -0.3177 | Yes |
| 81 | LCLAT1 |  |  | 7658 | -0.089 | -0.3125 | Yes |
| 82 | P4HA1 |  |  | 7667 | -0.089 | -0.3010 | Yes |
| 83 | SNX14 |  |  | 7861 | -0.099 | -0.3093 | Yes |
| 84 | GSTO1 |  |  | 7890 | -0.101 | -0.2985 | Yes |
| 85 | IFNGR1 |  |  | 7892 | -0.101 | -0.2845 | Yes |
| 86 | SERPINB6 |  |  | 7989 | -0.106 | -0.2807 | Yes |
| 87 | AHNAK |  |  | 8006 | -0.107 | -0.2676 | Yes |
| 88 | ADAM19 |  |  | 8040 | -0.110 | -0.2561 | Yes |
| 89 | ITGA6 |  |  | 8043 | -0.110 | -0.2410 | Yes |
| 90 | BMPR2 |  |  | 8138 | -0.117 | -0.2355 | Yes |
| 91 | PRNP |  |  | 8140 | -0.117 | -0.2193 | Yes |
| 92 | IGF2R |  |  | 8211 | -0.122 | -0.2103 | Yes |
| 93 | CYFIP1 |  |  | 8224 | -0.124 | -0.1944 | Yes |
| 94 | TWSG1 |  |  | 8229 | -0.124 | -0.1776 | Yes |
| 95 | CKAP4 |  |  | 8298 | -0.132 | -0.1670 | Yes |
| 96 | ALCAM |  |  | 8323 | -0.135 | -0.1509 | Yes |
| 97 | NT5E |  |  | 8380 | -0.144 | -0.1373 | Yes |
| 98 | CD81 |  |  | 8625 | -0.201 | -0.1373 | Yes |
| 99 | EMP1 |  |  | 8641 | -0.209 | -0.1099 | Yes |
| 100 | COL6A1 |  |  | 8698 | -0.238 | -0.0831 | Yes |
| 101 | CD44 |  |  | 8778 | -0.337 | -0.0452 | Yes |
| 102 | SPP1 |  |  | 8779 | -0.338 | 0.0020 | Yes |
Table: GSEA details [plain text format]

  

Fig 2: HALLMARK\_IL2\_STAT5\_SIGNALING: Random ES distribution      
 Gene set null distribution of ES for **HALLMARK\_IL2\_STAT5\_SIGNALING**
